# Nectar Robbing Positively Influences the Reproductive Success of *Tecomella undulata* (Bignoniaceae)

**DOI:** 10.1371/journal.pone.0102607

**Published:** 2014-07-18

**Authors:** Vineet Kumar Singh, Chandan Barman, Rajesh Tandon

**Affiliations:** Department of Botany, University of Delhi, Delhi, India; Centro de Investigación y de Estudios Avanzados, Mexico

## Abstract

The net consequence of nectar robbing on reproductive success of plants is usually negative and the positive effect is rarely produced. We evaluated the influence of nectar robbing on the behaviour of pollinators and the reproductive success of *Tecomella undulata* (Bignoniaceae) in a natural population. Experimental pollinations showed that the trees were strictly self-incompatible. The three types of floral colour morphs of the tree viz. red, orange and yellow, lacked compatibility barriers. The pollinators (*Pycnonotus cafer* and *Pycnonotus leucotis*) and the robber (*Nectarinia asiatica*) showed equal preference for all the morphs, as they visited each morph with nearly equal frequency and flower-handling time. The sunbirds caused up to 60% nectar robbing, mostly (99%) by piercing through the corolla tube. Although nectar is replenished at regular intervals, insufficient amount of nectar compelled the pollinators to visit additional trees in bloom. Data of manual nectar robbing from the entire tree showed that the pollinators covered lower number of flowers per tree (5 flowers/tree) and more trees per bout (7 trees/bout) than the unrobbed ones (19 flowers/tree and 2 trees bout). The robbed trees set a significantly greater amount of fruits than the unrobbed trees. However, the number of seeds in a fruit did not differ significantly. The study shows that plant-pollinator-robber interaction may benefit the self-incompatible plant species under conditions that increases the visits of pollinators among the compatible conspecifics in a population.

## Introduction

Nectar robbing is an outcome of the ability of some floral foragers to steal nectar without effecting pollination [1], [2]. The phenomenon is prevalent in many taxonomically unrelated species of flowering plants, and particularly those that hold concealed nectar in a tubular or spurred corolla [2]–[4]. The robbers may either pierce through the corolla tube [1], or make a hole in the calyx cup [5] to withdraw nectar. Interestingly, the robbers may sometimes change their role to pollinators in the same or different plant species [4].

Nectar robbing carries an obvious negative connotation with detrimental outcomes on the fitness of plants [2], [6], [7]. However, there are several instances where robbing produces partial-negative or weak-positive effects [8]–[10]. The net positive consequence becomes apparent when the fruit-set increases in response to robbing [11], [12]. Although the phenomenon of nectar robbing is of common occurrence [3], the evidences for the types of interaction-frameworks and the key attributes that yield a range of consequences, have only recently begun to emerge [4], [13], [14].

In a plant-pollinator-robber interaction milieu, the dynamics of nectar production and pollinator's behaviour have a crucial bearing on the net outcome [13], [15]. Nectar robbers can directly affect the plant reproductive success either by damaging the floral parts [16], [17] or by acting as pollinator in the same or different floral visit [8], [18]. Indirectly, the robbers may benefit sexual reproduction of plants by altering the behaviour of pollinators [19], [20].

The net consequences of nectar robbing are largely realized in the context of plant mating system. Whereas nectar robbing usually produces a negative to weak-positive effect in self-compatible systems, in self-incompatible (SI) plants increased pollen flow between the conspecifics may yield greater positive effects provided that the pollinator is not limited [13], [21]. Additionally, the pollinator's shorter flower-handling time and fewer floral visits per plant in a bout may also reduce the chances of stigma clogging by self-pollen [22]. Owing to several problems in designing the experiments, these aspects have been rarely investigated in tree species. There are also relatively limited studies that represent the effect of avian nectar robbers in ornithophilous self-incompatible plant species [7], [23], [24].

Obligate self-incompatibility [25]–[27] and nectar robbing are frequently encountered in Bignoniaceae [12], [28]. Also, the robbers and pollinators may belong to the same or different functional group(s) in a pollination guild. In addition to having large and showy flowers, production of copious amount of sugar-rich nectar is a key reproductive investment in the bignons. Nectar is released by a massive nectary disc located around the base of ovary and accumulates in the corolla tube.

We selected a natural population of *Tecomella undulata*, a tree bignon, to test the effect of nectar robbing on its reproductive success. As a prerequisite, we investigated the functional floral morphology, dynamics of nectar production and the mating system of the plant species, before integrating the interaction variables (robbers and pollinators) in the study. The study demonstrated that nectar robbing positively influences the reproductive fitness of *T. undulata* by influencing the foraging pattern of the pollinators.

## Material and Methods

### Species studied and study site

*Tecomella undulata* (Bignoniaceae), popularly known as the Desert Teak, is a medium-sized (6-10 m in height) deciduous tree. The trees grow naturally in the desert tracts of Western India. Slow growth rate and plundering of wood for making furniture are threatening the natural populations of the trees in the wild [29]. There are three colour morphs of *T. undulata* viz. red, yellow and orange, which are known to lack breeding barriers [30].

The study was conducted in a natural population with ∼200 trees located at Barmer, Rajasthan, Western India (25°35′68″ N; 71°14′71″ E). *Acacia tortilis*, *Prosopis cineraria*, *Salvadora oleoides*, *Capparis decidua* are the other trees in the community while most of the ground vegetation is covered by *Crotalaria burhia.* The flowering period of the plant spans between the third week of January and the fourth week of March, and is the only flowering species in the community at that period of time.

The field study was permitted by the Office of the Principal Conservator of Forests and the Chief Wildlife Warden, Forest Department, Jaipur, Rajasthan (permit no. 3(05)TK-11/PCCF/2010/7264). The study was not carried out in a protected area or on the protected species. Also, the study did not involve collection of any of the animals.

### Mating system

To establish the mating system, the trees were randomly selected (n = 24 trees, 8 of each morph). After identifying the peak receptive stage of stigma with peroxidase test [31], we randomly applied four pollination treatments- (i) spontaneous autogamy (n = 80 flowers), flowers were bagged one day before anthesis without emasculation; (ii) facilitated autogamy (n = 130 flowers), self-pollinated with pollen from the same flower or tree and bagged; (iii) xenogamy (n = 130 flowers), flowers were emasculated and pollinated with pollen grains from a different tree; (iv) apomixis (n = 80 flowers), flowers were emasculated and bagged. The open-pollinated flowers (n = 130 flowers) were considered as control.

### Natural Pollination Efficiency

Pollination efficiency (pollen deposition on stigma after first visit of the pollinator [31]) was determined from a random set of 20 flowers. The stigma was stained and mounted in 0.2% auramine O and the total amount of pollen deposition on the stigma was scored by using an epifluorescence microscope (Zeiss Axioscope A1, Germany).

### Floral visitors

The floral visitors, foraging frequency and flower-handling time were recorded during the peak time of blooming in staggered periods of 30 min each, every two hours for 10 days between 0600 h and 1800 h for the diurnal visitors, and from 1900 h to 2300 h (n = 3 nights) for the nocturnal visitors. For this, each time, a suitable patch of intensely flowering trees was identified and the observations were made with the aid of a pair of binoculars (Nikon, SMZ 800). Flower-handling time was recorded by using a digital stopwatch. The foragers were identified as pollinators when they legitimately consumed nectar and their body came in contact with anthers and stigma. The illegitimate foraging behaviour (nectar consumption by piercing through the corolla tube) was considered robbing.

### Forager's perception of the three morphs

As colour polymorphism in flowers may influence the extent of foraging [7], [32], it was crucial to determine that whether or not any of the three colour morphs had a significant influence on the foraging pattern of each bird type. We computed the (i) flower-handling time and (ii) number of flowers visited, by each type of bird on each type of randomly marked tree morph (n = 12 trees, 4 of each morph). The differences among the tree morphs were measured through MANOVA. The flower-handling time and frequency of visits were considered dependent variables; the three tree colour morphs and the three birds (robbers and pollinators) were considered as fixed factor. As neither of these parameters showed significant differences among the three morphs, the three morphs were considered as one unit for further experiments. Among birds differences were analysed through post-hoc Tukey test.

### Proportion of robbed flowers

It was not possible to record nectar thieving (nectar removal from the throat of corolla tube without effecting pollination), as the robbers might have visited the flower earlier than the time of observation. Therefore, percentile nectar robbing was determined by recording the number of flowers with pierce marks in their corolla tube, in randomly selected trees (n = 15 trees, each morph).

### Dynamics of nectar production

Nectar production in a flower begins by 0500 h and by the time the anthesis begins (0530 h onwards), the flowers are full of their first nectar crop. The average amount of nectar consumed by the pollinators and robbers was separately computed by deducting the average amount of nectar left after their first consumption from the mean fresh nectar crop (n = 42 flowers).

The amount of nectar and its sugar concentration, available to the nectar foragers in the replenishment phase, was determined by the method of Castellanos [33]. The flowers were randomly bagged (with fine mosquito net) 16–18 h before anthesis. After removing the first crop of nectar, one set of these flowers (n = 5 flowers of each morph) was subjected to hourly extraction (0800 h to 1400 h) of nectar with a calibrated syringe (5 ml), for a total of 6 h duration; the flowers were re-bagged after each extraction. For control, all the available nectar was collected after 6 h from the other set of flowers (n = 5 flowers of each morph). The cumulative amount of nectar replenished and the corresponding averaged values of sugar concentration were noted down. The total amount of sugars in nectar of each extraction was determined with the aid of a hand-held refractometer (0–80%, Sigma). The differences in cumulative replenished volume and the mean sugar concentration among three morphs were analysed through two-way ANOVA; the morphs and treatments were considered fixed factors. As the three morphs did not differ either in the amount of nectar replenished or the mean sugar concentration at each of the extraction hours, the data for three morphs were pooled for plotting the graphs.

### Foraging behaviour

First, we noted down foraging period of floral visitors (robbers and pollinators) through direct observations at regular intervals of one hour each (0700 h to 1800 h), on two consecutive days of peak flowering. Second, we recorded the total number of trees visited by pollinators over a period of one week in response to the available nectar crop at each hour. The average amount of nectar crop available at corresponding hours was measured from a set of randomly tagged flowers (n = 15 each hour).

The pollinator avoidance of robbed flowers was ascertained from a pair of manually robbed (with syringe) and unrobbed flowers (n = 20 flower pairs) on randomly selected trees (n = 5 trees). The number of visits was recorded between 0800–1400 h.

### Effect of nectar robbing on foraging behaviour

The effect of nectar robbing on pollinators and fruit-set was established by comparing the outcomes of manually robbed flowers with those that were prevented from robbery by applying a cello-tape [7], [34]. As the trees were medium-sized, it was possible to either use a collapsible bamboo ladder or climb the trees to approach the flowers; the trees were randomly selected in two different locations in the population, each manned by two people for recording the observations. To mimic natural robbing, nectar was extracted with a syringe (2 ml) by gently piercing it through the corolla tube of all the fresh and unrobbed flowers available in a tree before the arrival of robbers (n = 8 trees; n = 1296 flowers); the older flowers were removed. In order to prevent robbing, the lower part of the floral tube with nectar was collared with cello-tape (1.5 inches wide) in equal number of trees (n = 8 trees, n = 1120 flowers). Subsequently, three variables were recorded - (i) the flower-handling time (ii) the total number of flowers visited by the birds per tree, and (iii) the total number of trees visited by pollinators per bout. The effect of this treatment on pollinator behaviour was analysed through MANOVA. As the flowers tend to replenish nectar, the data were confined up to the first two hours (0800 to 1000 h) of the foraging period of pollinators.

### Effect on fruit and seed-set

In order to determine the effect of nectar robbing on reproductive success (% fruit and seed-set per fruit), the flowers of robbed and unrobbed trees were bagged and tagged soon after the first visit of pollinators. The tagged flowers were then monitored for fruit and seed-set. The difference in fruits and seed-set between robbed (n = 8 trees) and unrobbed trees (n = 8 trees) was estimated by one-way ANOVA; trees were considered as fixed factors. Additionally, fifteen fruits each were randomly collected from the robbed and open-pollinated trees to compare the difference in average number of seeds set in a fruit by using one-way ANOVA. The mean ovule production was determined by dissecting the unpollinated pistils (n = 20 flowers).

Data analyses were carried out with SPSS 16 statistical software (SPSS Inc. 2007). Percentage data were root-square arcsine-transformed. The values are presented as mean ± standard error.

## Results

### Mating system

Controlled pollinations showed that there was no fruit-set either through spontaneous or facilitated autogamy. All the selfed flowers abscised within 4 days of pollination and only the cross-pollinated flowers (xenogamy) developed into fruits. Flowers bagged to ascertain apomixis also failed to set fruits. Thus, the species exhibited obligate xenogamy. Fruit-set from the cross-pollinated flowers was significantly greater (t = 8.49, df = 258, *P* = 0.001, two sample t-test) than the open-pollinated ones (Table 1). The natural pollination efficiency was 367.65±20.29 pollen grains per stigma, which was sufficient to fertilize all the ovules (312.83±6.25) in a pistil.

**Table 1:** Results of experimental pollinations in *T. undulata*.

Pollination treatments	% Fruit-set (Flowers pollinated)
Spontaneous autogamy	0 (80)
Facilitated autogamy	0 (130)
Xenogamy	**84.62 (130)** **
Apomixis	0 (80)
Open-Pollinated (Control)	38.46 (130)

** P<0.001.

### Floral visitors and the foraging behaviour

**Figure 1:**
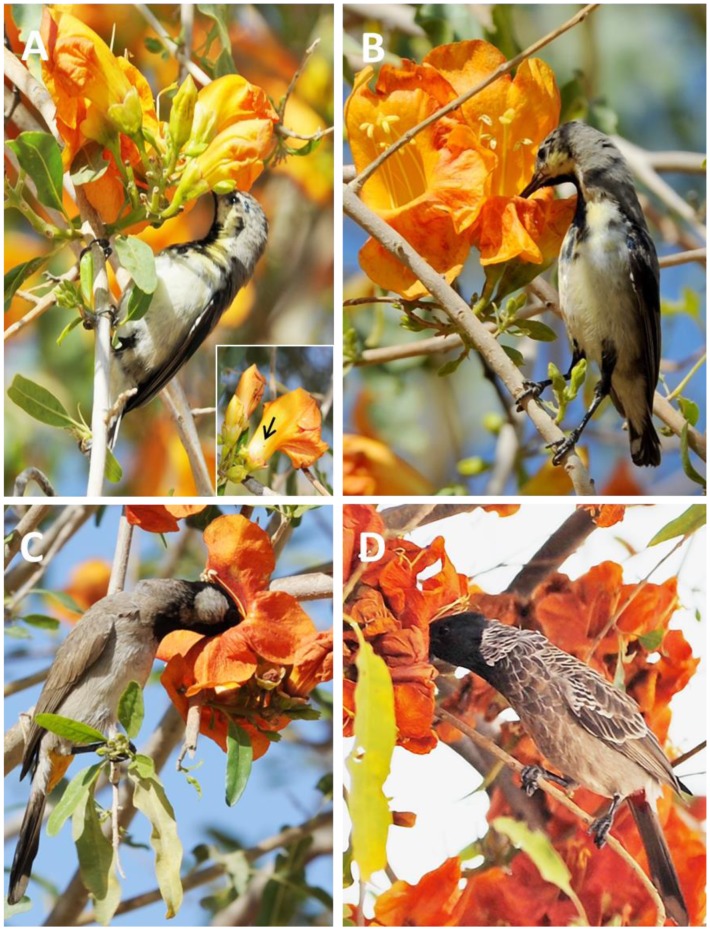
Robber and pollinators of *T. undulata*. (A) The robber (*Nectarinia asiatica*) consuming nectar by piercing through the corolla tube, and (B) through the opening of the tube. (C & D) The two pollinators - *Pycnonotus leucotis* and *Pycnonotus cafer* legitimately forage the flowers and facilitate pollination. Inset of (A): Note the hole (arrow) in the corolla tube made by the robber.

The bright showy flowers attracted the birds (Table 2), particularly two species of bulbul, the red-vented bulbul (*Pycnonotus cafer*) and the white-cheeked bulbul (*Pycnonotus leucotis*) and one species of sunbird (*Nectarinia asiatica*) (Fig. 1A–D). Both the bulbuls legitimately foraged the flowers for nectar and were able to bring about pollination in the species. As their beaks are shorter than the sunbirds, bulbuls foraged deep through the corolla tube. Pollen grains were transferred from their nape/crown when they made contact with the dehisced anthers and the stigma (Fig. 1 C,D).

**Table 2:** Details of floral visitors in *T. undulata.*

Species	Peak time of Visitation	Flower-handling time (sec.)
**Nectar Robber**		
*Nectarinia asiatica*	0900–1000	1.6±0.09 (n = 65)
**Pollinator**		
*Pycnonotus cafer*	1000–1100	3.5±0.18 (n = 68)
*Pycnonotus leucotis*	1000–1030	2.7±0.18 (n = 63)

The purple sunbirds (including their females) robbed the flowers of nectar mostly (∼99%) by piercing through the corolla tube and rarely (1%) by consuming nectar from the opening of the tube, without coming in contact with the stigma or anthers (Fig.1 A,B).

### Forager's perception of the three morphs

**Figure 2:**
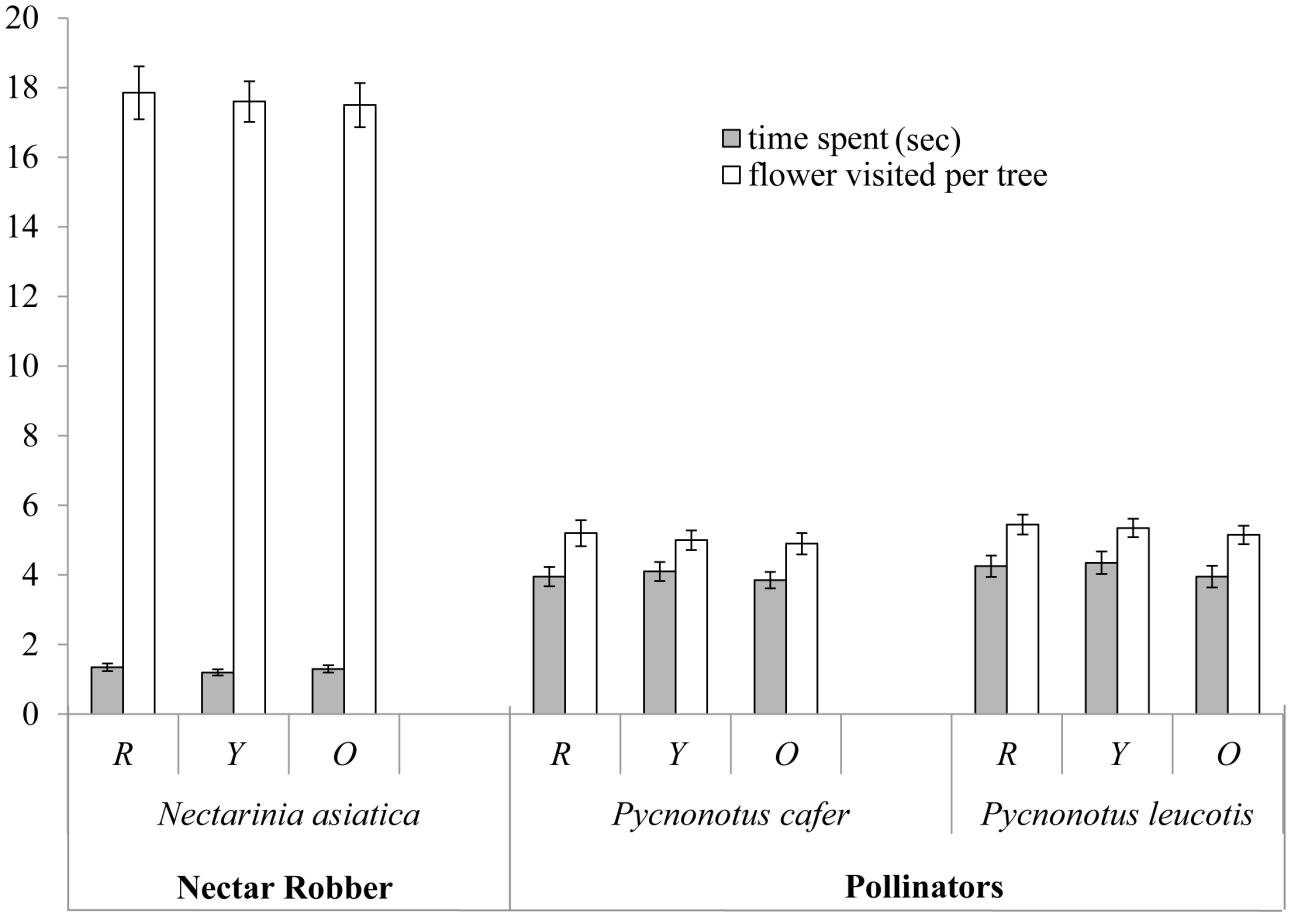
Foragers perception of the three morphs (R/Y/O, red/yellow/orange tree morphs). The robbers spent significantly less time and visited a greater number of flowers than the pollinators.

The three colour morphs of the tree were perceived as one by the foragers, because the three morphs did not differ significantly (*F*_(4,340)_ = 0.42, p>0.05; Wilk's λ = 0.99, partial η^2^ = .005, MANOVA) in terms of the flower-handling time (total observations, n = 180) and the frequency of visits (total observations, n = 180). However among birds, significant difference were seen (*F*_(4,340)_ = 204.27, p = 0.001, Wilk's λ = 0.086, partial η^2^ = 0.70, MANOVA). The two bulubuls species responded equally (Fig. 2) and visited fewer flowers with longer flower-handling time in a tree than the purple sunbirds (post-hoc Tukey HSD).

### Proportion of Nectar Robbing

Number of flowers with pierce marks did not differ among the three morphs (F = 1.310, df = 2,42; *P* = 0.05, one-way ANOVA); the pierce marks in the flowers of orange, yellow and red tree morphs were 66.2% (n = 2565 flowers), 63.8% (n = 2299) and 60.5% (n = 1947), respectively.

### Nectar production and replenishment

**Figure 3:**
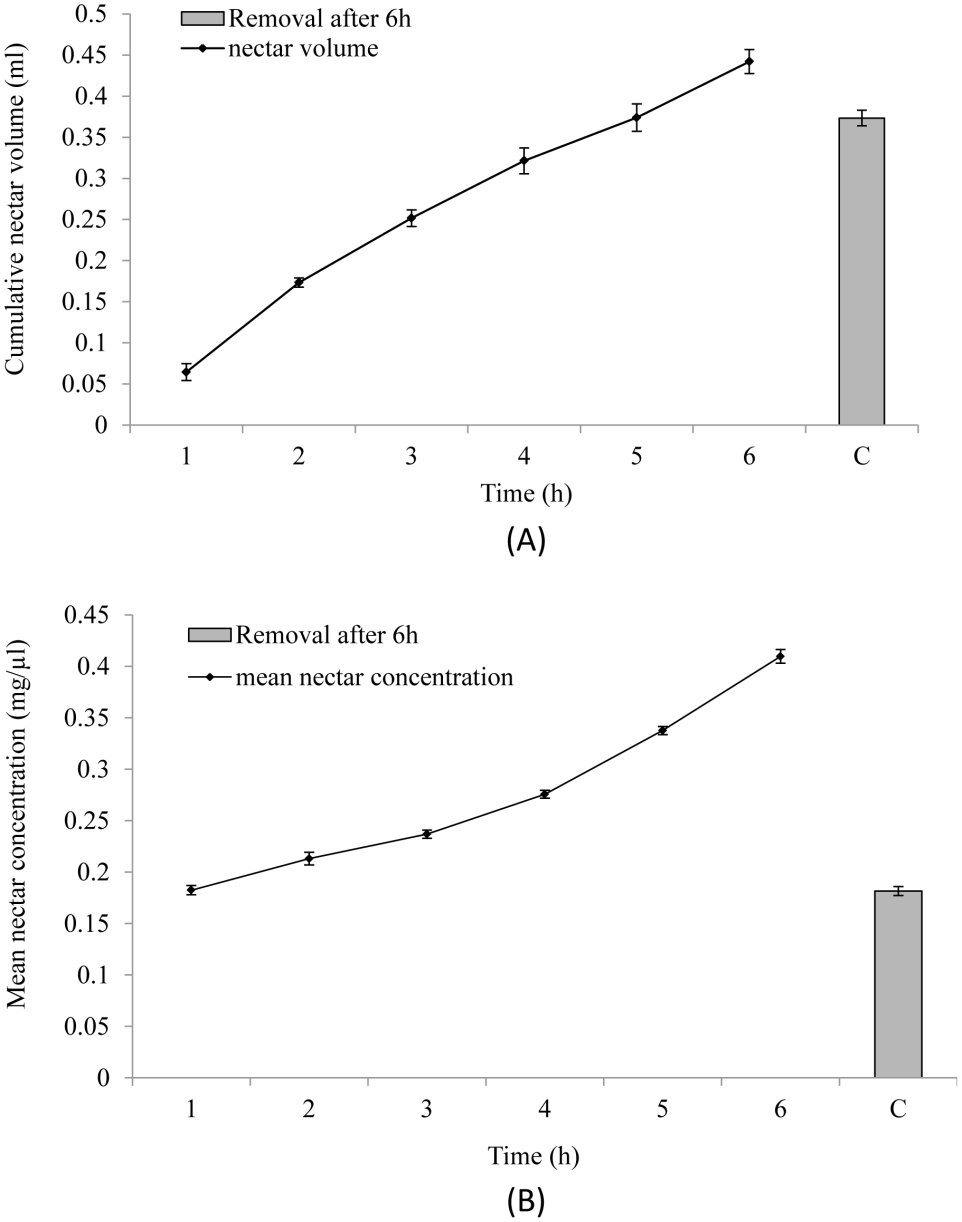
Nectar replenishment and sugar concentration (averaged in the three morphs) in response to extraction. A. Cumulative nectar replenishment after hourly extraction of nectar. B. Mean nectar sugar after hourly extraction of nectar. Histogram on the right of each figure represents the respective values of single extraction after 6(C). The bars indicate standard error.

The average standing crop of nectar before robbing was 0.51±0.02 ml and after the first round of robbing, it declined to 0.2±0.01 ml. Thus, on an average the robbers consumed ∼0.3 ml of nectar from a flower (n = 42 flowers). On the other hand, the average amount of nectar consumed by a bulbul was 0.42±0.01 µl (n = 20, unrobbed flowers). Removal of nectar facilitated its replenishment in the flowers. The three morphs did not differ in the amount of nectar replenished (*F*_2,24_ = 0.009, *P* = 0.991, two-way ANOVA) and the mean sugar concentration (*F*_2,24_ = 0.485, *P* = 0.622, two-way ANOVA). The cumulative amount of replenished nectar from the multiple extraction was greater than that from the single extraction after 6 h (*F*_1,24_ = 34.43, *P* = 0.001, two-way ANOVA, Fig. 3A). Similarly, the mean sugar concentration was significantly different from the single extraction (*F*_1,24_ = 170.58, *P* = 0.001, two-way ANOVA, Fig 3B).

### Foraging behaviour

The robbers began foraging 40–60 min (at 0700 h) prior to the arrival of the pollinators. During this gap, nearly ∼60% of the freshly opened flowers (n = 15 trees) in a tree had been robbed of their first flush of nectar. The pollinators visited the robbed flowers without any discrimination from the unrobbed ones (Wilcoxon singed-rank test: *Z* = 0.447, *P* = 0.766).

The robbers visited 19.82±1.01 flowers (n = 60 observations) in a tree and 2.8±0.13 trees (n = 30 robbers) per bout within the population, while the pollinators foraged 9.87±0.64 flowers (n = 120 observations) within a tree and 7.55±0.42 trees (n = 60 pollinators) in a bout. During the second peak phase of foraging, both the pollinator birds visited fewer trees (3.02±0.19) and more flowers per tree (15.75±0.74) than the first phase (8.29±0.42, trees and 4.49±0.21 flowers per tree).

**Figure 4:**
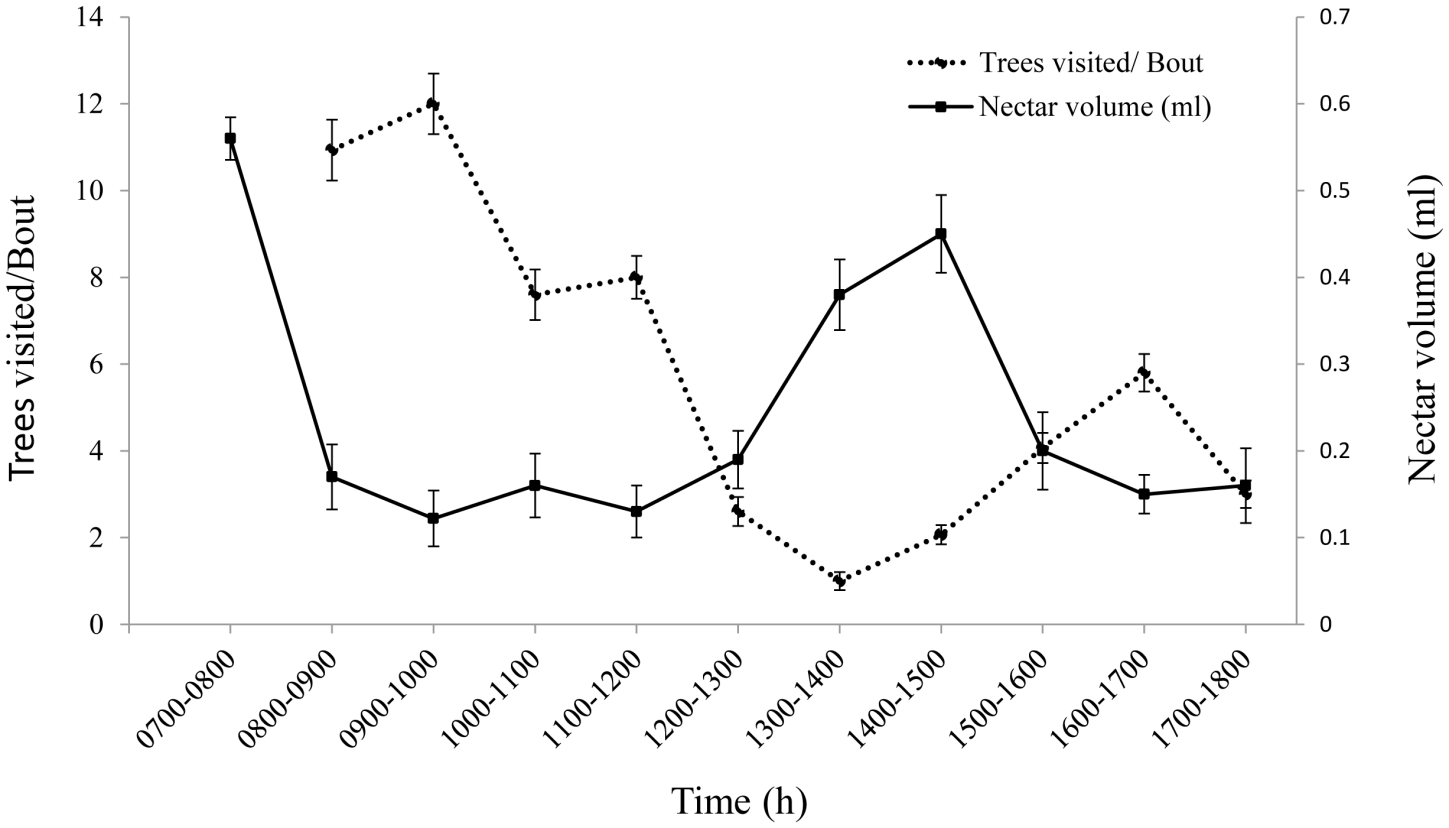
Foraging pattern of pollinator in response to nectar production. Nectar availability and the number of tree visits in a bout by the pollinators correlate negatively in its bimodal distribution pattern.

The foraging pattern of the birds was distinctly bimodal in distribution with two peak phases of foraging (Fig. 4); first between 0800 and 1000 h and the second between 1500 and 1700 h. The number of trees visited in a bout by the pollinators correlated negatively (y = 0.5835x, r^2^_adj_ = −0.773) with the nectar crop available at different times in a day.

### Effect of nectar robbing

**Figure 5:**
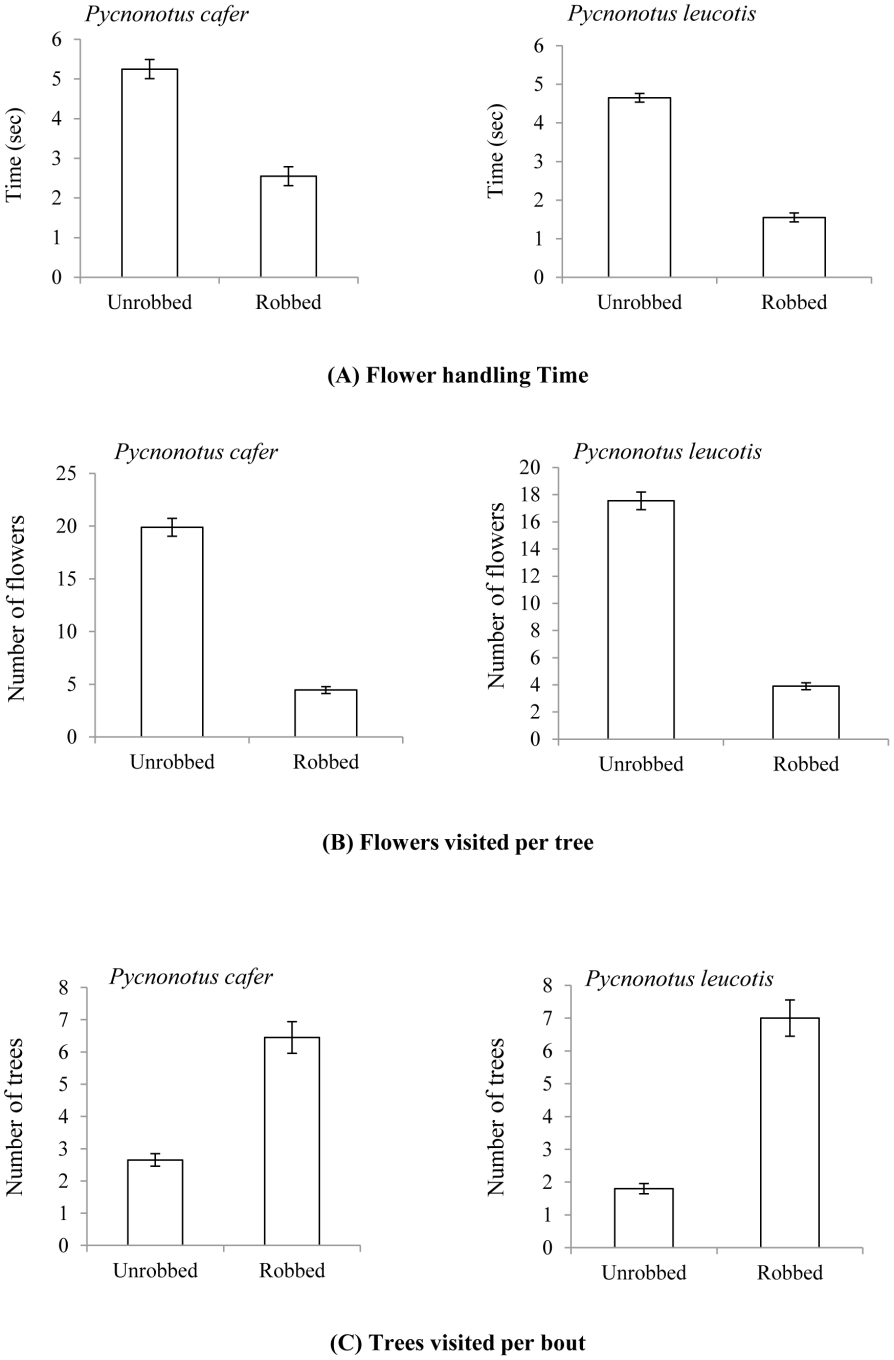
Foraging behaviour of the two pollinators on experimentally robbed and unrobbed trees. Whereas (A) the flower handling time and (B) the number of flowers visited per tree declined upon nectar removal from all the flowers in a tree, the number of trees covered per bout (C) by both the pollinators was increased.

Collaring of the floral tubes in the unrobbed trees did not deter either the pollinators from foraging the flowers normally, or prevented the robbers from making an attempt to rob the flowers of nectar in a usual manner. The nectar robbing treatment significantly influenced (F _(3,74)_ = 296.9, p>0.001; Wilk's λ = 0.007, partial η^2^ = 0.923) the behaviour of pollinators in terms of the three recorded parameters viz. (i) flower handling time, (ii) flowers visited per tree and (iii) trees visited per bout. The flower-handling time and the number of flowers visited per tree by the pollinators were significantly lower in robbed trees (2.05±0.16 sec; 4.17±0.29 flowers/tree; n = 20 of each pollinator) than the unrobbed ones (4.95±0.23 sec; 18.72±0.75 flowers/tree; n = 20 of each pollinator) (Fig. 5). Also, robbing treatment in a tree led to an increase in the number of subsequent tree visits in a foraging bout in the robbed trees (6.72±0.5, n = 20 of each pollinator) as compared to the unrobbed ones (2.22±0.17; n = 20 of each pollinator) (Table 3). Although the two bulbuls did not differ in terms of the number of trees visited in a bout, the red-vented bulbul spent significantly more time on a flower and visited more flowers per tree than the white-eared bulbul (Table 3).

**Table 3:** Effect of experimental nectar robbing on pollinator behaviour in *T. undulata*.

Source	Flower-handling time		Floral visits/tree		Trees visits/bout	
	ss	F	ss	F	Ss	F
Treatments (robbed vs unrobbed)	168.20	205.52^**^	4234.05	641.65^**^	405.0	**133.42** ^**^
Pollinators (*P. cafer* vs *P. leucotis*)	12.80	15.64^**^	42.05	6.37^*^	0.450	0.148
Treatments* Pollinators	0.80	0.98	16.20	2.45	9.800	3.228

Tests of between-subject effects for the (i) flower handling time (sec.), (ii) the number of flower visited per tree and (iii) number of trees in a foraging bout of pollinators, in response to experimental nectar robbing. df = 1,79. ^*^ = 0.01<p<0.05; ^**^ = p<0.01; F =  F ratio; ss  =  sum of squares.

Whereas fruit-set among the robbed and unrobbed trees differed significantly (*F* = 8.30, df = 1,15; *P* = 0.012, one-way ANOVA), the mean number of seeds in a fruit did not (*F* = 0.145, df = 1,15; *P* = 0.709, one-way ANOVA). Also there was no difference in the extent of seed-set in a fruit between the robbed and the open-pollinated trees (*F* = 0.15, df = 1,29; *P* = 0.904, one-way ANOVA). The mean ovule production was 288.45±7.34 (n = 20 pistils) and the seed to ovule ratio was 0.573±0.02 (n = 20).

## Discussion

The present work demonstrates that under a set of circumstances, nectar robbing may impart a strong positive influence on the reproductive success of the species. In obligate self-incompatible *Tecomella undulata*, nectar robbing is neither destructive to the flowers nor reduces the opportunity for cross-pollination. Robbing promotes indiscriminate foraging by the pollinators in the robbed and unrobbed flowers and the trees morphs, which promotes fruit set.

### Mating system

Experimental pollinations showed that fruit-set is realized only through cross-pollinations (xenogamy). The occurrence of absolute self-incompatibility in the trees is in agreement with the other obligate outbreeding bignoniaceous trees such as *Jacaranda rugosa* [28], *Oroxylum indicum* [27], *Spathodea camapnulata* [25] and *Tabebuia nodosa* [35].

In strictly outbreeding trees including the bignons, natural fruit set is usually low (<5%) [27], [28], [36], [37], and pollination success is essentially dependent on the efficacy of pollinators to bring compatible pollen [38], [39]. In most of these species including *T. undulata* (present work), experimental cross-pollinations yielded greater fruit set than the open-pollinations, suggesting limitation of cross-pollination. In this context, nearly 40% natural fruit-set with multi-seeded condition (∼165 seeds per fruit) in *T. undulata* is appreciably high, which suggests an intense pollinator activity. In the absence of any other co-flowering species in the vicinity, *T. undulata* was the only floral resource for the birds. A copious and solitary floral resource in the community probably confined the interaction dynamics of the birds, in their nesting period, to a single plant species in bloom. As the natural pollen load on the stigma was sufficient to fertilize all the ovules in a pistil, it is likely that an intense foraging by the pollinators might be resulting in mixed self-(through geitonogamy) and cross-pollination in the species. The twofold increment in fruit-set from the experimental cross-pollinations indicates the effect of application of pure cross pollen.

### Pollination and foraging pattern

*Tecomella undulata* exhibits a typical ornithophilous pollination syndrome [40]. The flowers are large, nectar-rich and showy with trumpet-shaped corolla tubes serve as strong visual cues to attract the birds. Among these bulbuls were effective in pollination as they foraged legitimately through the floral opening and dimensionally there was a morphological compliance between the nape or crown of the bird and opening of the floral tube. In many plants species, besides legitimacy in foraging, a morphological match between the phenotypes of flower and the pollinators is a prerequisite for success in pollination [27], [40]. The nonconforming foragers, such as the purple sunbirds in *T. undulata*, were rendered as nectar robber/thieves.

In addition to the quantity of nectar, its quality may also influence the foraging behaviour of pollinators [2], [41]. Among the two peak foraging periods observed in the study, the forenoon peak had greater number of pollinator visits per bout. The difference could be attributed to prior foraging by robbers (∼1 h) in the first peak (between 0800–1100 h) that led to the conditioning of the behaviour of pollinators to visit additional flowers. Before the arrival of pollinators, ∼60% of the freshly opened flowers were robbed of their first nectar crop, thereby promoting a greater number of visits of pollinators per bout. However, during the second peak, the replenished nectar crop was simultaneously consumed by the three bird species. The availability of a surplus amount of nectar confined the pollinators to spend more time in a tree, as the energy requirements are fulfilled from a fewer flowers. Also, as the day passes, nectar becomes more viscous and sucrose rich than its first crop, which might have impeded swift consumption of the reward from several flowers.

### Effect of nectar robbing on fruit and seed set

The results of the experimentally manipulated trees were in accordance with the direct observations. The positive influence of robbing emerged from significantly greater fruit-set in experimentally robbed trees than the controls. Also, the pollinators were not limited in the population, which ensured sufficient visitation to the flowers. This is in contrast to several other species where robbing results in low fecundity due to pollinator limitation [13], [42], [43].

Interestingly, seed-set in a fruit did not differ between the experimentally robbed and unrobbed trees indicating that even shorter flower-handling time ensured sufficient pollen deposition on the stigma. As pollination in these trees was natural, the proportion of seeds is most likely to be similar to the natural scenario. In spite of sufficient pollination, the lower seed set (seed number per fruit) than the ovules could be due to low amount of ovule receptivity i.e. the readiness of the mature ovules to receive pollen tubes [44]. Low ovule receptivity has been reasoned for lower seed-set than the ovule number in *O. indicum*, an obligate self-incompatible bignon [27].

Nectar removal from the flowers stimulates its replenishment at an additional energy cost to the plants [2], [4], [45]. The cost is increased if pollinators avoid patch of robbed flowers [12], [46]. The extra cost of nectar production may get balanced with chance revisits of pollinators to the previously robbed flowers and also, if the pollinators do not avoid the robbed flowers [4], [13], [47]. In *T. undulata*, the robbed flowers were repeatedly replenished with nectar subsequent to each robbing and the positive effects become assured when the pollinators visit both the robbed and unrobbed flowers and the three morphs without any discrimination. Thus, the tendency of pollinators to not to avoid the robbed flowers could be considered as one of the potential predictable attributes in ascertaining the net effects of nectar robbing in plants.

Nectar robbing has an indirect and positive influence on the sexual reproduction of *T. undulata*. The benefit of this interaction (increased fruit set) appears to outweigh the possible negative effect such as pollen discounting through geitonogamy. The circumstances under which the benefits are accrued include the inability of the pollinators to avoid the robbed flowers, replenishment of nectar and the foraging behaviour of the pollinator subsequent to robbing. Robbing engenders a trap-lining behaviour in the pollinators with enhanced inter-tree visits in the population, which promotes the required outbreeding. Thus, the robber plays a constructive and crucial role in the reproductive performance of this threatened tree species. It has been suggested that a plant may continue to sustain robbing provided that its pollinators do not become a limiting factor [48]. In *T. undulata*, there are two pollinators at the site with nearly equal performance and the robbers indirectly facilitate cross-pollination by strongly integrating into the pollination system.

Usually, the benefits and costs are not static in a plant-pollinator mutualistic interaction and may vary with species composition in a community or seasons [49]. Although our study does not highlight the seasonal variation in the interaction pattern at the site, the pattern may unlikely deviate, considering the (i) strong seasonal phenology of the trees that matches with the diversity and availability of nectar feeding birds in the region, (ii) requirement for legitimacy by suitable birds to effect pollination, and (iii) obligate self-incompatible mating system of the plant species. However, the pattern may likely change under conditions where sympatric coflowering ornithophilous species may compete for the pollinator birds.
